# Murine Metatarsus Bone and Joint Collagen-I Fiber Morphologies and Networks Studied With SHG Multiphoton Imaging

**DOI:** 10.3389/fbioe.2021.608383

**Published:** 2021-06-11

**Authors:** Martin Vielreicher, Aline Bozec, Georg Schett, Oliver Friedrich

**Affiliations:** ^1^Institute of Medical Biotechnology, Department of Chemical and Biological Engineering, Friedrich-Alexander University Erlangen–Nürnberg, Erlangen, Germany; ^2^Department of Internal Medicine 3 – Rheumatology and Immunology, University Clinic, Erlangen, Germany

**Keywords:** collagen-I fibrillar networks, collagen fiber morphology, bone microstructure, multiphoton SHG microscopy, bone and joint diseases, remodeling 3D Morphology

## Abstract

Chronic inflammatory disease of bones and joints (e.g., rheumatoid arthritis, gout, etc.), but also acute bone injury and healing, or degenerative resorptive processes inducing osteoporosis, are associated with structural remodeling that ultimately have impact on function. For instance, bone stability is predominantly orchestrated by the structural arrangement of extracellular matrix fibrillar networks, i.e., collagen-I, -IV, elastin, and other proteins. These components may undergo distinct network density and orientation alterations that may be causative for decreased toughness, resilience and load bearing capacity or even increased brittleness. Diagnostic approaches are usually confined to coarse imaging modalities of X-ray or computer tomography that only provide limited optical resolution and lack specificity to visualize the fibrillary collagen network. However, studying collagen structure at the microscopic scale is of considerable interest to understand the mechanisms of tissue pathologies. Multiphoton Second Harmonic Generation (SHG) microscopy, is able to visualize the sterical topology of the collagen-I fibrillar network in 3D, in a minimally invasive and label-free manner. Penetration depths exceed those of conventional visible light imaging and can be further optimized through employing decalcification or optical clearing processing *ex vivo*. The goal of this proof-of-concept study was to use SHG and two-photon excited fluorescence (2-PEF) imaging to mainly characterize the fibrillary collagen organization within *ex vivo* decalcified normal mouse metatarsus bone and joint. The results show that the technique resolved the fibrillar collagen network of complete bones and joints with almost no artifacts and enabled to study the complex collagen-I networks with various fiber types (straight, crimped) and network arrangements of mature and woven bone with high degree of detail. Our imaging approach enabled to identify cavities within both cortical and trabecular bone architecture as well as interfaces with sharply changing fiber morphology and network structure both within bone, in tendon and ligament and within joint areas. These possibilities are highly advantageous since the technology can easily be applied to animal models, e.g., of rheumatoid arthritis to study structural effects of chronic joint inflammation, and to many others and to compare to the structure of human bone.

## Introduction

Bone is a highly complex cellular-matrix composite structure with self-healing and auto-regeneration capabilities of smaller volumetric defects. Bone contains various different cells including bone forming osteoblasts, bone degrading osteoclasts as well as their respective precursor cells and osteocytes. The bone marrow in cancellous bone acts as host and breeding site for immune cells (Moreira et al., 2019). The extracellular matrix of bone is very complex and a major key for its diverse biomechanical features (Saini et al., 2019). It is built from collagen-I fiber and fiber bundle networks which are adsorbed to hydroxyapatite (HAp) nanocrystals formed by biomineralization (Dorozhkin, 2011; Murshed, 2018). The cooperation of these two elements is correlated for bone strain resistance with remaining flexibility and ultimate compression stability (Quesada et al., 2006; Shoulders and Raines, 2009; Alford et al., 2015).

**FIGURE 1:**
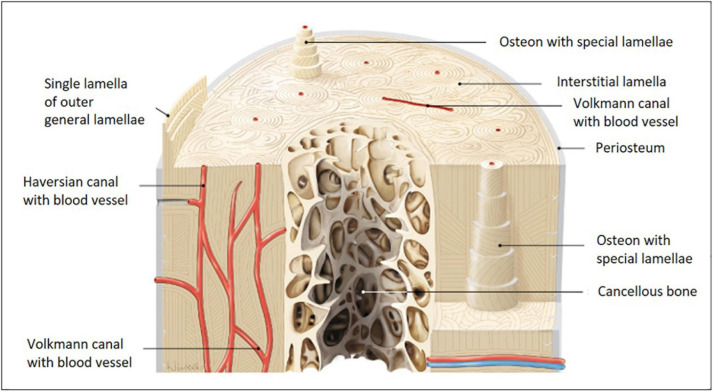
Models of long bone anatomy. Human long bone anatomy at the diaphysis level shown on a cross and transverse section model. The cortical bone sheet is made up of osteons with Haversian and Volkmann canals. Osteons with special lamellae surrounding the Haversian canals are visible in detail in the model. The lamellae are formed from sheets of aligned collagen-I fibers and fiber bundles, which are gradually shifted in an oriented manner. Their substructure is very characteristic and is built from fibers with alternating orientations (image adapted from Paulsen and Waschke 2010).

Distinct zones with specific structural designs are found within bone. Cortical bone comprises a dense and strong fibrillary collagen network located underneath the periosteum. Here and in cancellous bone, collagen-I fibrils are highly ordered as straight longitudinal bundles. Along the Haversian canals, these fibrils are organized in lamellae (Figure 1, canals together with lamellae are referred to as Haversian systems). Within each lamella, fibers organize in parallel, but the orientation varies in neighboring lamellae with respect to the long axis of the bone (Burke et al., 2017). This layer-wise formation is very characteristic and being reorganized during development, growth, traumatic injury, chronic inflammatory, or degenerative disease. Haversion system remodeling is known to be different between species, e.g., humans and rodents (Weiner and Traub, 1992; Jilka, 2013; Alford et al., 2015; Theocharis et al., 2019). Small mammals, such as rats and mice, do not possess Haversian systems, because their bones are small enough to permit adequate nutrient, waste and signal exchange for embedded osteocytes, therefore, blood vessels within Haversian canals are not required.

Various different collagen-I fiber types and networks are found within long bones. The structural design of the collagen meshwork varies between the diaphysis and epiphysis zone due to different functionality and mechanical load on these areas. The same is true for the metaphysis zone (growth plate) in between, where *de novo* formation of (woven) bone occurs. Due to the close structure-function-relationships usually found in the body, it seems quite clear, that the micro-structure of collagen-I networks will vary between the different types of bones found within the body. Due to its high density and thus, optical scattering properties, imaging bone can be challenging. This is why bone requires decalcification respectively optical clearing pre-processing to perform deep-tissue imaging in the best possible manner (Alers et al., 1999; Begum et al., 2010; Kim and Bixel, 2020).

The currently most preferred tool to monitor and quantify 3D bone formation is μCT scanning. However, multiphoton Second Harmonic Generation (SHG) microscopy is gaining importance as it enables depth-resolved, high resolution imaging of the fibrillar collagen networks without the need for labeling (Cox et al., 2003; Zheng et al., 2009; Zimmerley et al., 2010; Saitou et al., 2018; Zitnay et al., 2018). SHG scattering signals from collagen together with two-photon excited fluorescence (2-PEF) from cells and other extracellular matrix constituents provide valuable complementary information on bone structure. Due to the fact, that SHG imaging is a least-invasive technique, artifacts could solely derive from fixation and eventually decalcification, which warrants that fiber structures and morphologies are close to the native state. It is very beneficial that SHG almost exclusively detects collagen-I and hardly any other fibrous components within bone. The SHG signal is not specific to collagen-I at the molecular level. However, at the macromolecular level it depends on the organization and assembly into microfibrils, fibrils, and fibers. In collagen-rich tissues like bone the SHG signal intensity is directly correlated with the packing density and degree of alignment of the collagen fibrils, as well as with their relative orientation with respect to the imaging plane and polarization of the excitation beam (Campagnola et al., 2002; Stoller et al., 2003; Williams et al., 2005; Erikson et al., 2007; Chen et al., 2012; Tuer et al., 2012; Cicchi et al., 2013). High SHG intensities are indicative of dense assemblies of well aligned fibrils with the same polarity, essentially lying in the plane of observation, while a lower signal can result from either (or both) a less-organized collagen matrix or an out-of-plane fibril orientation.

Multiphoton imaging (MPI) can be used to study collagen organization in healthy and diseased tissue, development and aging, in particular in the fields of bone/fracture healing, joint/cartilage, tendon and ligament, and normal connective tissue research (Caetano-Lopes et al., 2010; Rahmati et al., 2017). Collagen-I fiber networks in bone or bone-related tissues have been studied with SHG imaging in a variety of settings. Most of these studies used animal models of osteoarthritis [human, (Brown et al., 2013), mouse (Caetano-Lopes et al., 2010; Mingalone et al., 2018; Saitou et al., 2018)], osteogenesis imperfecta (mouse, Nadiarnykh et al., 2007), intervertebral disk injury (mouse, Reiser et al., 2007) or induced metastasis (rat, Burke et al., 2017) to study changes in collagen-I fiber framework in bone or cartilage tissues. Other studies performed on bovine (Chaudhary et al., 2015) or equine (Mansfield and Peter Winlove, 2012) tissues were focusing on age-dependent collagen network changes. Human cortical bone (knee and articular cartilage) was studied by Houle et al. (2015) and its shear deformation and fracture by Tang et al. (2015).

Another category of studies targeted toward comparing SHG imaging with other non-linear techniques (e.g., CARS), with SEM or TEM or histological examination (Caetano-Lopes et al., 2010; Mansfield and Peter Winlove, 2012; Capitaine et al., 2018) or applied new models for quantitative analysis of collagen fiber networks (Reiser et al., 2007; Deniset-Besseau et al., 2010; Brown et al., 2013; Chaudhary et al., 2015; Saitou et al., 2018). SHG imaging was used to assess local periodic structures, orientation, morphology, diameter, and bundling of collagen fibers in bone or cartilage (Brown et al., 2013; Forouhesh Tehrani et al., 2020; Pendleton et al., 2020) and for example to discriminate cartilaginous from osseous tissues or to detect structural disorder (Saitou et al., 2018).

The fibrillar collagen network in mouse bone (femoral cortical and trabecular bone and metatarsal tendon, bone and bone marrow) was studied by SHG imaging in Rakhymzhan et al. (2020) in which the authors introduced a dual approach (optical coherence tomography and two-photon microscopy) to fill the gap between fundamental science and clinical small scale tissue imaging approaches. In another study (Genthial et al., 2017), third-harmonic generation (THG) imaging was applied to study porosity and interfaces within bone of several species (bovine, human, ewe, mouse) including mouse (femur full cortical transverse sections). Their results show that the porous lacuno-canalicular network of bone could be analyzed in relation to the fibrillar collagen matrix organization by simultaneously recording THG and SHG signals.

The aim of this paper was to use SHG and 2-PEF imaging to characterize the fibrillary collagen organization within *ex vivo* decalcified normal mouse metatarsus bone and joint. In most studies murine bone was used (often together with bone of other species) to introduce new imaging methods like OCT, THG, CARS, among others, often in combination with SHG to image the collagen network in a more global manner. Other studies used murine bone mainly as a study object for more advanced quantification assays of collagen amounts, organization and polarization, often on a theoretical level. In only a very small number of studies, the structure of the collagen network in murine bone was investigated in more detail and compared to the human counterpart. Our data provide evidence for a heterogenous organization of the fibrillar collagen network with a variety of different fiber morphologies. This enables a better understanding of mouse bone structure as a starting point to better compare normal to diseased bone in animal models.

## Materials and Methods

### Preparation and Decalcification of Mouse Metatarsus Bone

Metatarsus bone (length: 5 mm) was isolated from the paw of a six months old C57/BL6 mouse (wildtype, *n* = 3) as described in Oláh et al. (2016). The long bones were prepared in Ringer’s solution by removing neighboring bones and all surrounding muscle and tendon tissue. As MPI quality of untreated bone was poor due to the dense, opaque bone matrix still containing inorganic HAp, decalcification was required. We used an acidic 10% EDTA solution (pH 5) to mildly decalcify the long bone from HAp at room temperature to improve optical access to the collagen-I matrix within. After one day, bright, dot-like structures still appeared as 2-PEF signals, which we interpreted as HAp crystals. Therefore, we continued decalcification for a total of four days using mild agitation (three times per day). The resulting bones were opaque and flexible (could be easily bent using a forceps).

### Preparation and Decalcification of the Mouse Metatarsus Joint Region

Eight weeks old, non-arthritic male mice (strain: 129/B6) used in studies of arthritis development in joints (Hannemann et al., 2019) were chosen for this study. Their joints in the metatarsus region are usually inflamed after induction for arthritis. The paws (18 mm length, *n* = 3) were from mice which were not induced for arthritis. These were skinned, fixed overnight in 4% para-formaldehyde, washed in PBS, and stored in 70% ethanol. After removing surrounding muscle, tendon and ligament structures, the metatarsus region was dissected using a stereomicroscope. Following this, sample decalcification was performed for four days as described in the section “Preparation and Decalcification of Mouse Metatarsus Bone.”

### Multiphoton Imaging

Multiphoton imaging of collagen-I networks was described in detail elsewhere (Vielreicher et al., 2018). Briefly, we used a TriMScope II (LaVision Biotec, Bielefeld, Germany), a high-performance inverted, multiphoton microscope equipped with a femtosecond (fs)-pulsed Ti:Sa laser with dispersion precompensation and tunable wavelengths in the range 710–980 nm. All images were collected using a water immersion LD C-apochromat objective (×40/NA 1.1/UV-VIS-IR/WD 0.62; Zeiss, Jena, Germany) and detected with high-sensitivity GaAsP photomultipliers (PMTs) mounted in non-descanned configuration close to the back aperture of the objective. Two-photon excitation occurred at 810 nm and signals were separated by a dichroic mirror at 460 nm. Wavelengths >460 nm were detected as 2-PEF, shorter wavelengths were reflected and blocked with a 405/20 bandpass filter (SHG in backscattering mode from fibrillary collagen-I). Prepared samples were illuminated from below (inverse system). Merged 2-PEF/SHG images of cells/collagen-I were generated using Fiji image processing and analysis software (NIH, Bethesda, MD, United States).

#### Imaging of an Isolated Metatarsus Long Bone

The murine metatarsus long bones were positioned in glass-bottom dishes (thickness: 170 μm, filled with PBS buffer) above the objective. Imaging was performed at the hypophyseal and epiphyseal zone (pixel dwell time: 0.5 μs). The imaged z range in both regions was >100 μm. Various regions within the different zones of the metatarsus long bone were imaged and the presented images show the typical morphologies in the best manner. Sample variability was low.

#### Imaging of the Dissected Metatarsus Joint Region

The dissected metatarsus joint area was positioned in close proximity to the surface of a glass-bottom dish (filled with PBS buffer) which was placed above the objective to enable MPI from below. Images with a size of 200 μm × 200 μm were recorded (1,024 × 1,024 pixel, pixel dwell time: 0.7 μs). Z-projections images were calculated in Fiji from the average intensity of (varied number of) images from the z-stack to show the global organization or a certain z-region of the fibrillar collagen network. For larger field-of-view imaging, 2D mosaic image series (8 × 8 images) were taken with an image overlap of 6% using Imspector software (LaVision Biotec). Image stitching was performed using the Autostitcher Plugin of Fiji. The size of the resulting mosaic image was 1,136 μm × 1,136 μm (8,121 × 8,121 pixel). Various regions within the metatarsus region were imaged and the provided images represent typical examples. Sample variability was low.

## Results

### MPI of Collagen-I Fiber Morphologies in Isolated Mouse Metatarsus Long Bone

**FIGURE 2:**
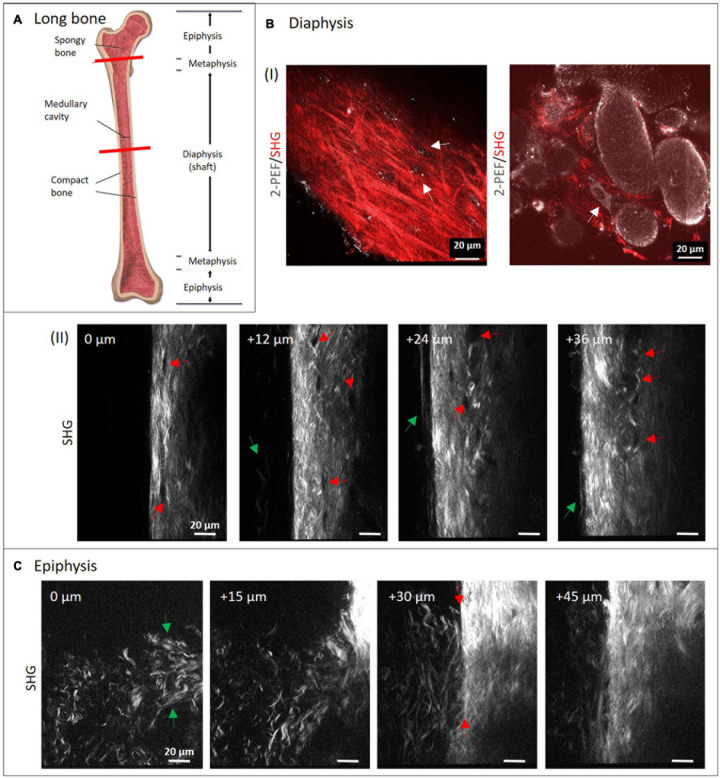
Multiphoton imaging through long bone diaphysis and epiphysis. **(A)** Anatomy of a long bone with epiphysis and diaphysis zone. Image from Blausen (2014). Creative Commons Attribution License (http://creativecommons.org/licenses/by/4.0) Decalcified mouse metatarsi were imaged using multiphoton SHG imaging at increasing depths. In panel **(B)** images from the diaphysis zone are shown (950 × 1,400 pixels, 133 μm × 196 μm). In panel **(I)**, dual-color images (1,429 × 1,429 pixels, 200 μm × 200 μm) highlight 2-PEF (gray) and SHG signals (red) from cortical and cancellous bone, whereas in panel **(II)**, a series of SHG images from cortical bone is presented. In panel **(II)**, red arrows direct toward rounded areas with no signal, green arrows point toward collagen-I fibers at the very periphery. In panel **(C)** recordings from the epiphyseal zone are presented (1,200 × 1,400 pixels, 168 μm × 196 μm).

Typical long bone comprises of diaphysis and epiphysis zone with a metaphysis zone in between on both sides as depicted in the scheme in Figure 2A. Bone is covered entirely with periosteum and compact, cortical bone encloses the cancellous (or spongy) bone located in the center. We performed MPI of mouse metatarsus long bone at the diaphyseal (Figure 2B) and the epiphyseal zone (Figure 2C; the imaged region is marked in Figure 2A by red lines).

Applying a successful decalcification procedure made it possible to dissolve almost all inorganic HAp and to image through the whole bone structures (>100 μm) where we collected high resolution images throughout. HAp crystals are highly light-diffracting and the cause for the high opacity of bone. They usually appear as autofluorescent spots with a characteristic, sharply defined shape. The chosen conditions of decalcification were effective as those autofluorescent spots completely disappeared during the course of incubation. Collagen-I fibers and HAp crystals are known to be tightly interconnected in bone. Nevertheless, HAp removal by decalcification ahead of imaging the collagen network is a common procedure used in many other studies. In a SHG imaging study by Saini et al. (2019) it was furthermore demonstrated that complete removal of HAp by EDTA treatment did not affect collagen-I assembly.

The isolated long bone had to be decalcified with EDTA at lowered pH for four days. In both zones, the complete diameter (>100 μm) was imaged using SHG signals from fibrillary collagen (see also Supplementary Videos 1, 2). Images were taken with a z-distance of 3 μm. Much lower z-distances would have been possible to reconstruct 3D volume views, but were not within the scope of this study. Here, four representative images with 12 and 15 μm z-distance between images show how the structure patterns of fibrillary collagen changes in 3D in both zones. In the image series in Figure 2B-II, collagen-I appears as aligned fibers with different lengths and variable intensity of the SHG signal (see bright regions at 24 and 36 μm). This is a sign of a variable degree of fiber bundling or fiber densities. Their orientations appear quite diverse on all images of the series and are clearly different from the degree of order as in human lamellar bone (model in Figure 1). At the periphery (left side), very long, loosened fibers (green arrows) are visible that appear to represent parts of the periosteum. Black rounded to ellipsoid structures (red arrows) are visible in all images, especially aligned in high density at 24 and 36 μm. At 0 μm, it appears that there is a connection between these regions from top to bottom (between the two red arrows). The two dual-color images in Figure 2B-I show SHG (red) together with 2-PEF (gray) signals. The left image is comparable to the images in Figure 2B-II. Here, the collagen-I fibers next to fiber bundles, all with very diverting thickness and orientations became obvious. Autofluorescent dots (gray) are located in cavities where no collagen-I fibers are present (white arrows). In the combined 2-PEF/SHG image to the right, collagen-I fibers are present only in low quantity and they fill the spaces between large rounded to ellipsoid autofluorescent structures (∼20–80 μm size) with a rough surface. The white arrow in the image points toward a single adherent cell with a nucleus.

Figure 2C shows a series of images from the epiphysis zone at increasing depth. Characteristic fiber morphologies are discernible, where evenly distributed crimped fibers and fiber sheets (green arrows at 0 μm) are visible between 0 and 30 μm. This area is neighbored by a zone with straight fibers of significantly higher fiber density (at 30 μm, right; and at 45 μm). In the image at 30 μm depth, the interface becomes visible very sharply (between two red arrows). Interestingly, crimped and straight fibers mix up at the left side of the line between the two arrows. Altogether, the image series is taken exactly at the transition between these two structural zones with obviously different functionality.

### MPI of Collagen-I Networks in Dissected Mouse Metatarsus Region

**FIGURE 3:**
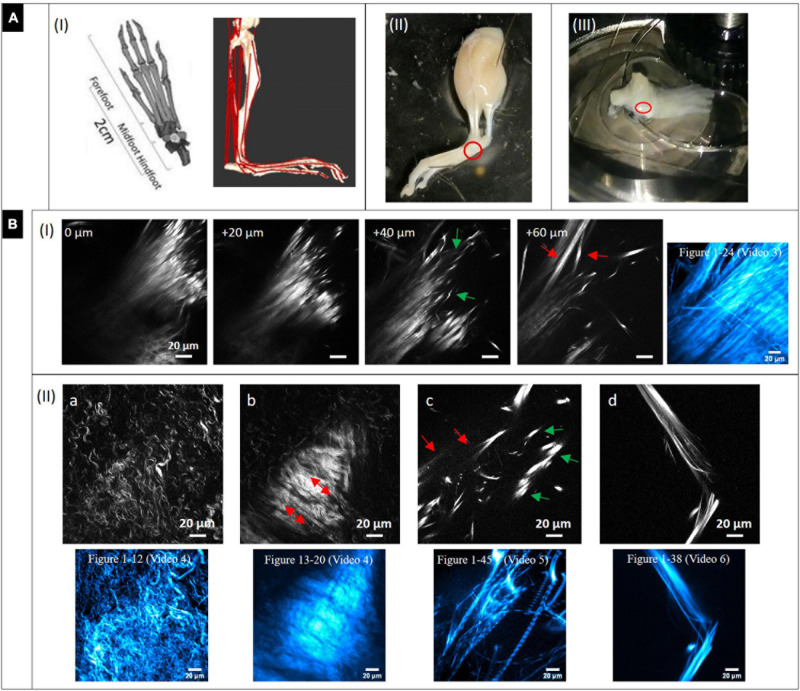
Dissection of mouse paw and multiphoton SHG imaging after decalcification. **(A)** Mouse paw. **(I)** Anatomy of mouse foot and ankle. **(Left)** Graphical depiction of skeletal model (transverse plane view, from Gao et al., 2019). Creative Commons Attribution License (http://creativecommons.org/licenses/by/4.0). **(Right)** Musculoskeletal model of a mouse’s right hindlimb (craniolateral view) developed with biomechanics software (from Charles et al., 2016 section of Figure). Musculo-tendon units (red) were incorporated into the model. The image shows a multitude of tendon structures running through the metatarsus region. **(II)** Skinned and PFA-fixed mouse foot. The metatarsus region is marked (red circle). **(III)** The lower foot was isolated and all muscle tissue on the sole entirely dissected, then it was placed in a glass bottom dish on top of the objective to optically access the region of interest (red circle). **(B)** Multiphoton SHG images from the metatarsus region (200 μm × 200 μm, 1,024 × 1,024 pixels). **(I)** Images series of a region of interest at increasing depths and **(II_a-d)** images of various regions showing areas with very different collagen-I fiber morphologies and arrangements. Z-projections of the complete image sequences (images in cyan) show the global organization of collagen network (compare to Supplementary Videos). Images **(a,b)** are from the same stack.

Figure 3A-I (left image) shows models of the murine foot and metatarsus region (midfoot). Various tendon and ligament structures run through this region (red lines in right image). In Figure 3A-II, a whole skinned mouse foot is shown. In order to expose the tiny metatarsus region (marked by red circle), all muscle and tendon tissue had to be dissected from the sole of the foot. To gain optical access, the dissected region was placed on a glass bottom dish (Figure 3A-III) for MPI from below. The SHG signals in the images in Figure 3B highlight fibrillary collagen (fibrils, fibers and fiber bundles, mostly from collagen-I) and show the diversity of morphologies, arrangements and packing found in structures within this region. The colored images (cyan) shown in addition represent z-projections from image stacks taken in the respective locations (with given numbers of images).

A series with images taken in 20 μm z-distance is shown in Figure 3B-I. The fibers have a straight morphology and are highly assembled and orientated in parallel. At their endings they become crimped (0 and 20 μm) and lacerate (40 μm, green arrows). At 60 μm, the large fiber bundle splits up into smaller, long bundles oriented into various directions (red arrow). These signals appear to originate from tendon and ligament structures. The z-projection shows the global distribution of fibers over a range of 24 images (depth of 240 μm). Supplementary Video 3 gives an impression of how the collagen network (SHG, red) and the fluorescence from cells (gray) is distributed in 3D.

In Figure 3B-II single SHG image snapshots are presented showing the various fiber morphologies that were found in the metatarsus region. Both Figures 3B-IIa,b were taken from the same image stack (same x–y location), but from a different depth. Figure 3B-IIa shows an area with collagen-I fibers, which are all in a crimped and curly morphology comparable to the ones found in epiphysis of bone (Figure 2B). There is almost no larger scale fiber assembly present. Instead, fibers are all equally fine-structured throughout and appear to show into the same direction (top-left to bottom-right). Figure 3B-IIb highlights an interface area that was located around 30 μm apart in z. A highly assembled and dense array of collagen-I fiber bundles is shown that was oriented in parallel with high SHG signal intensity and orientation from lower right to upper left (red arrows). Around this zone, collagen fibers exhibit the curly morphology visible in Figure 3B-IIa. The associated z-projections were taken from images ranging over a z distance of 40 μm in –Figure 3B-IIa (12 images) and 28 μm in (eight images, Δz: 3.6 μm) in –Figure 3B-IIb. Supplementary Videos 4 shows only the SHG channel. In Supplementary Video 4b, where the SHG channel is presented together with the 2-PEF channel, the location of cell arrays becomes clear very nicely in the second part of the video sequence (left side). Figure 3B-IIc shows an area with very bright, almost dot-like signals (green arrows) and much less bright fibrous structures (straight and aligned) in the background (red arrows). This area is comparable to the image series in Figure 3B–I (+40 μm) and appears to show the comparable crimped, lacerated fiber endings of straight fiber bundles. The z-projection below ranges over 45 images and impressively shows how the signal is oriented globally over a depth of 450 μm. The collagen network is quite loose in this area which is impressively underlined by Supplementary Video 5. An almost perfectly aligned, narrow-banded, straight fiber array (diameter only around 20 μm) is presented in Figure 3B-IId (compare to Figure 3B-I at 60 μm). Here, the z-projection image (38 images ranging over 190 μm with Δz = 5 μm) support the impression that this structure is highly ordered in 3D, like tendon or ligament. Supplementary Video 7 shows another z-stack from an additional region, in which the distribution of the 2-PEF signal (gray) of cells differs markedly from collagen network (SHG in red). Supplementary Video 8 (SHG) shows aspects of a network of crimped collagen in high spatial resolution.

**FIGURE 4:**
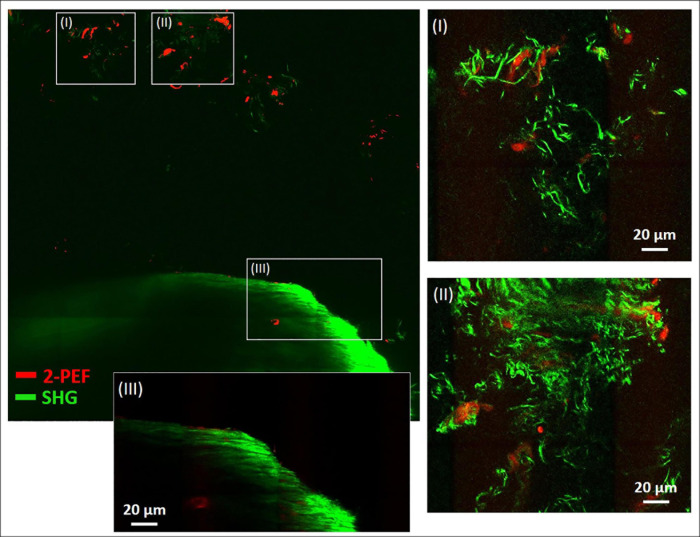
Combined multiphoton 2-PEF/SHG large-scale image of decalcified metatarsus region of mouse. Overview image (8 × 8 images, 1,136 μm × 1,136 μm) with enlarged regions **(I–III)** adapted for brightness and contrast. In panels **(I)** and **(II)** the collagen-I network is highly resolved with various fiber morphologies and arrangements. In panel **(III)** the periphery of the large object is enlarged and shows rather uniform fiber morphology typical for cortical bone. 2-PEF: cells/remaining HA crystals. SHG: collagen-I fiber networks.

In Figure 4, a large-area mosaic image of the dissected metatarsus region is shown, that was obtained from 2-PEF/SHG images (8 × 8). In the image, two different areas were observed, a smaller, isolated area with very low SHG signal intensity [enlarged in (I) and (II)] at the top and a large, filled object (bottom) with very intense SHG signal and almost no 2-PEF signal (III). In (III), the periphery is well-resolved (outer zone of ∼40 μm thickness) which contains by far the highest signal intensity. The signal appears to consist of densely organized straight collagen-I fibers reminiscent of the arrangement in the diaphysis of the metatarsus long bone in Figure 2A. After adjustment for brightness and contrast, area (I) and (II) becomes much better resolved. In both of these images, significant autofluorescent signals (2-PEF, red) are present as blurry shapes of varied size underlying the SHG signals of the collagen fiber network (green). In (I), solely crimped fiber morphologies with very low density are visible. In image (II), the fiber density is considerably higher and sheets of crimped parallel fibers are present in addition (upper part of image).

## Discussion

Our goal was to use SHG and 2-PEF imaging to characterize the fibrillary collagen organization within *ex vivo* mildly decalcified normal mouse metatarsus bone and joint. In this proof-of-concept MPI study, we focused on capturing the fine structure of the collagen fiber assemblies and networks present in different locations within the mouse metatarsus region to get a detailed picture of their microanatomy. Autofluorescent images taken in parallel to SHG images were helpful for interpretation of the observed collagenous structures, e.g., 2-PEF from cells located in bone cavities or blood vessels acted as landmarks for orientation. In this context, it is worth mentioning a study by Genthial et al. (2017), in which THG was used for morphological imaging, which allows to image interfaces and pores in bone, which are very well suited to provide orientation at small length scales. It would certainly be interesting to revisit this approach as well with mouse metatarsus.

The differences in collagen-I fiber arrangement in the diaphysis and epiphysis become obvious in Figure 2. In the diaphysis (cortical bone), solely straight, aligned fibers were present. The spatial orientation of the fibers was very diverse and disordered (Figure 2B-I, left). As already mentioned, mice lack fully developed Haversian systems. In order to still warrant stability with remaining flexibility, the collagenous fibers typically arrange in a way that was described as plywood motif (Weiner and Traub, 1992). Compared to this, human or large animal bone, collagen fibers are arranged in lamellae around Haversian canals in a high degree of order, which was shown by combined 2-PEF/SHG imaging in Tang et al. (2015) and (Li et al., 2019). This also becomes impressively clear in Genthial et al. (2017), where bovine, mouse, ewe and human bone was analyzed with THG. While Haversian systems were present in all the bone samples of higher animals, in mouse bone they were absent (Figures 5d,f). The higher degree of organization of the collagen network in mouse bone compared to our observations could be explained due to the fact, that transverse sections of much larger femur long bones were used by these authors.

In the image series of Figure 2B-II, rounded black zones (red arrows) with no SHG signal were found along and across the bone long axis, which actually seem to be empty areas. However, in the 2-PEF/SHG image (Figure 2B-I, left) bright 2-PEF signals (white arrows) were detected there, which we think have a cellular origin and may derive from either endothelial cells from blood vessels or remaining cells from osteocyte lacunae. Even though the chosen prolonged decalcification procedure completely dissolved HAp crystals, such treatment would not entirely wash out all cells, especially after fixation. Therefore, the roundish areas most likely represent cross-sectioned blood vessels running through bone, considering their high numbers and typical diameters (∼10 μm). The shapes of these black areas vary from roundish to ellipsoid. This fits well with our assumption that blood vessels run through murine metatarsal bone in a much more disorganized manner compared to human long bone, where their spatial organization is largely determined by the longitudinal direction of Haversian canals (Figure 1; Tang et al., 2015).

The image in Figure 2B-I (right) appears to represent a region within cancellous bone. By size, the large rounded autofluorescent structures would fit well with osteoclasts, however no nuclei were observed (osteoclasts are multinucleated cells). This becomes very clear in comparison with the single adherent cell (white arrow in image), which likely represents an osteocyte. By size, the large structures would also fit with fat bodies, however, their rough surface is rather untypical for fat bodies. The SHG signal, which is located in between these large autofluorescent structures, directs toward trabecular collagen-I after dissolution of the inorganic phase (HAp matrix), even though its morphology appears to differ somewhat from the one in cortical bone. We therefore interpret the large, rounded structures as remaining cavities in between trabeculae. We did not find another study that shows these structures in high resolution without labeling artifacts.

In contrast to what we found in cortical bone at the diaphysis zone, very significant, crimped, thin fibers were detected in the epiphysis zone (Figure 2C). These appear mostly as single fibers with only a low partition of more assembled, thicker fibers. At the interface to cortical bone, both fiber types gradually mix up which is very nicely shown in the image series. These images were likely from the growth plate (metaphysis), which in the tiny mouse bone, is located very close to the epiphysis zone. Our interpretation is that the crimped collagen acts either as a preliminary stage for woven bone (Shapiro and Wu, 2019), which exhibits an irregular orientation of straight collagen fibers with a low degree of organization. Woven bone is rapidly produced by osteoblasts before it is remodeled to mature, aligned collagen fibers at a later stage. Alternatively, the curly shape of the crimped collagen-I fibers is caused by a different extracellular matrix composition in the metaphysis (leading to different biomechanical conditions), where collagen-I is accompanied with elastic fibers (Chow et al., 2014). In principle, tile-scan 3D imaging with low Δz (<0.5 μm) would allow to more precisely map the fibrillary collagen distribution within the entire metarsus and represent it in 3D.

Similar collagen-I fiber morphologies and organization were detected also in the dissected metatarsus region, but in addition, different structures were observed there as well (Figure. 3B). Fibrillary arrangements with straight, long and highly aligned fibers with a parallel organization and their shape changing in 3D like in Figure 3B-I and Supplementary Video 3 were not present in isolated bone. As many other anatomical structures are present in the metatarsus region as well (Figure 3A-I), we interpret them to originate from tendon or ligament, which may have remained after dissection. In Supplementary Video 3, cellular distribution (by their 2-PEF signal) can be observed quite clearly as well. An even higher degree of alignment with only slight splitting of bundles appears in Figure 3B-IId. The structure is compact (diameter ∼20 μm) and does not change much in 3D as becomes apparent already in the z-projection image (over a depth of 190 μm) and also in Supplementary Video 6 and likely represents tendon or ligament. A different view on loss of organization of the collagen bundle network becomes visible in Figure 3B-IIc. Both, thin fibers (<1 μm) and thicker fibers (>8 μm) become obvious, which appear at a length scale from very short (∼10 μm) to long (50 μm and above). This high variability does obviously not represent a region within tendon/ligament, but rather one where tendon or ligament connects to muscle or bone. Based on their shape and distribution, the fibers with very high signal intensity appear to derive from projections of fine tendon/ligament structures, in which the collagen fibers are densely packed to linear bundles. This would explain their high signal intensity, as the SHG signal significantly increases with assembly grade and density of collagen fibers. However, the global distribution of bundles has a low density, which becomes clear by the provided z-projection image over a range as high as 450 μm in depth.

Figure 3B-IIa shows an area, in which the collagen fibers are all in a crimped morphology comparable to the ones in Figure 2C (left, interface of epiphysis to diaphysis), however in a more detailed view. The assembly grade of the fibers is very low and fibers exhibit a quite homogenous morphology and anisotropic orientation (top-left to bottom-right). Due to this high degree of organization, these fibers seem to originate from an ordered population of collagen-I forming cells (osteoblasts), probably within the growth plate. Figure 3B-IIb, which is from the same image stack (further above in depth; appears earlier in Supplementary Video 4/4b) highlights an interface area of a highly assembled and dense, parallel array of crimped fiber bundles with high signal intensity flanked by a zone with less densely arranged fibers and lower signal intensity (left and right periphery of Figure 3B-IIb). The similarity to Figure 2C makes it likely that these images derive from the same region (interface of epiphysis to metaphysis in metatarsus bone). However, the crimped fiber morphology does not transition into a straight morphology (as in Figure 2-C), but remains wavy, only very densely packed. Supplementary Video 4b with additional 2-PEF channel provides information about the cellular distribution in this area.

Figure 4 provides a dual channel (2-PEF/SHG) large area view of the metatarsus region simultaneously showing cells and the fibrillary collagen network. The large structure at the bottom (III) filling almost the whole width of the tile-scan image (∼1 mm) contains a densely aligned fiber network with very high SHG signal intensity, especially at the periphery and likely is cortical bone. The collagen networks in areas (I) and (II), on the other side, are much less dense. SHG signal intensity is very low there due to a low degree of collagen fiber assembly and density. A likely explanation for these loosely arranged fiber networks in the area outside bone in a joint region is that they belong to fibrocartilage tissue, which is usually present in joints. Fibrocartilage is the only type of cartilage that contains fibrous collagen-I in various proportions (mediating inflexibility and toughness) in addition to the normal collagen-II (enabling elasticity). A similar kind of characteristically crimped and wavy collagen-I fiber arrangement was also reported for elastic tissues like aorta, arteries and annulus fibrosus in studies using SHG imaging (Wells, 2003; Chow et al., 2014). Altogether it also seems likely that in locations (also within bone), where the fibrillar collagen exhibits a crimped morphology, more flexibility is required. Interestingly, fibrocartilage tissue is also present in locations where tendons and ligaments attach to bone (Provenzano and Vanderby, 2006) and could be an explanation for the crimped collagen fibers and high variability observed in Figure 3B-IIc.

Concerning fibrillary collagen changes in disease, work with animal models has shown, how fiber networks are affected in arthritic inflammation and degradative processes in bone (osteoporosis, osteogenesis imperfecta, etc.) (Huber, 2007; Bonucci and Ballanti, 2014; Licini et al., 2019; Mobasheri et al., 2019; Shapiro and Wu, 2019). This aspect was recently studied by Mingalone et al. (2018), with SHG imaging of collagen changes in mouse knee articular cartilage in early osteoarthritis. Their results revealed, that in particular SHG was very well suitable to show the onset of the changes which included an increased proportion of thinner fibers and changes in tissue layer-dependent collagen fiber organization. Similar studies would be possible that focus on collagen structural changes in bone caused by disease or aging or development.

## Data Availability Statement

The raw data supporting the conclusions of this article will be made available by the authors, without undue reservation.

## Ethics Statement

Ethical review and approval was not required for the animal study because experiments were only performed in tissue harvested from animals sacrificed for research purpose and were in accordance with the local Animal Welfare guidelines and regulations regarding animal handling (project number: TS-6/2016). No live animal experiments were performed. Written informed consent was obtained from the owners for the participation of their animals in this study.

## Author Contributions

MV designed the concept, performed the imaging and analysis, and wrote the manuscript. AB and GS provided mouse samples (129/B6) and funding. OF co-conceived the study, proof-read the manuscript, and provided funding. All authors contributed to the article and approved the submitted version.

## Conflict of Interest

The authors declare that the research was conducted in the absence of any commercial or financial relationships that could be construed as a potential conflict of interest.
