# Supplementary material for: Murine Metatarsus Bone and Joint Collagen-I Fiber Morphologies and Networks Studied With SHG Multiphoton Imaging
Source: Front Bioeng Biotechnol. 2021 Jun 11;9:608383. doi: 10.3389/fbioe.2021.608383 (PMC8226188; doi:10.3389/fbioe.2021.608383)
Supplement: Supplementary file 10 [file Data_Sheet_1.PDF]

Supplementary Videos

Supplementary Videos are all in .avi format with 3 frames per second (3 fps) and show SHG or 2-PEF/SHG 3D image series of the regions presented in the manuscript (Figure 2A/B and 3B-a – 3B-e). Size bars are inserted in each image sequence.

Videos 1 and 2 show the imaged region presented in Fig. 2 as a complete 3D image stack (isolated metatarsus).

<u>Video 1.</u> Image stack (SHG) from the diaphysis region from Fig. 2B-II (z-range: 111 µm, z-distance between images (Δz): 3 µm).

<u>Video 2.</u> Image stack (SHG) from the epiphysis region from Fig. 2C (z-range: 102 µm, Δz: 4 µm).

Videos 3-6 are from the joint region presented in Fig. 3B.

<u>Video 3.</u> Dual color image stack (2-PEF in grey, SHG in red) from image series presented in Fig. 3B-I (z-range: 240 µm, Δz: 10 µm).

<u>Video 4.</u> Image stack (SHG) from image series presented in Fig. 3B-II-a and -b (z-range: 68 µm, Δz: 3.6 µm).
<u>Video 4b.</u> Like Video 4, but dual color stack (2-PEF in grey, SHG in red).

<u>Video 5.</u> Image stack (SHG) from region in Fig. 3B-II-c (z-range: 450 µm, Δz: 10 µm).

<u>Video 6.</u> Image stack (SHG) from region in Fig. 3B-II-d (z-range: 190 µm, Δz: 5 µm).

<u>Video 7.</u> Image stack (2-PEF in grey, SHG in red) from another region not shown in Fig. 3B (z-range: 270 µm, Δz: 10 µm).

<u>Video 8.</u> Image stack (SHG) from another region not shown in Fig. 3B (z-range: 147 µm, Δz: 3.5 µm).
